# Single Nucleotide Polymorphism Typing of *Mycobacterium ulcerans* Reveals Focal Transmission of Buruli Ulcer in a Highly Endemic Region of Ghana

**DOI:** 10.1371/journal.pntd.0000751

**Published:** 2010-07-20

**Authors:** Katharina Röltgen, Weihong Qi, Marie-Thérèse Ruf, Ernestina Mensah-Quainoo, Sacha J. Pidot, Torsten Seemann, Timothy P. Stinear, Michael Käser, Dorothy Yeboah-Manu, Gerd Pluschke

**Affiliations:** 1 Swiss Tropical and Public Health Institute, Molecular Immunology, Basel, Switzerland; 2 University of Basel, Basel, Switzerland; 3 Ghana Health Service, Ministry of Health, Tema, Ghana; 4 Department of Microbiology, Monash University, Clayton, Australia; 5 Victorian Bioinformatics Consortium, Monash University, Clayton, Australia; 6 Noguchi Memorial Institute for Medical Research, University of Ghana, Legon, Ghana; Institut Pasteur, France

## Abstract

Buruli ulcer (BU) is an emerging necrotizing disease of the skin and subcutaneous tissue caused by *Mycobacterium ulcerans*. While proximity to stagnant or slow flowing water bodies is a risk factor for acquiring BU, the epidemiology and mode of *M. ulcerans* transmission is poorly understood. Here we have used high-throughput DNA sequencing and comparisons of the genomes of seven *M. ulcerans* isolates that appeared monomorphic by existing typing methods. We identified a limited number of single nucleotide polymorphisms (SNPs) and developed a real-time PCR SNP typing method based on these differences. We then investigated clinical isolates of *M. ulcerans* on which we had detailed information concerning patient location and time of diagnosis. Within the Densu river basin of Ghana we observed dominance of one clonal complex and local clustering of some of the variants belonging to this complex. These results reveal focal transmission and demonstrate, that micro-epidemiological analyses by SNP typing has great potential to help us understand how *M. ulcerans* is transmitted.

## Author Summary

*Mycobacterium ulcerans* causes a destructive skin disease known as Buruli ulcer (BU), which has been reported from more than 30 tropical or subtropical countries, with the highest prevalence in Western Africa. Due to the striking genetic monomorphism of African *M. ulcerans* populations, conventional genetic fingerprinting methods have largely failed to differentiate isolates coming from the same BU endemic area. Here we report a highly discriminatory fingerprinting method for *M. ulcerans* using a single nucleotide polymorphism-based genetic fine-typing technique. This method has enabled us for the first time to identify different *M. ulcerans* haplotypes within a BU endemic area. Linking the origins of *M. ulcerans* strains with the patients' residences unveiled the clustering of unique *M. ulcerans* haplotypes within the Densu river basin of Ghana. Results show, that haplotypes do not spread within a short time over the entire BU endemic region, but rather form independent focal transmission clusters.

## Introduction

Infection with *Mycobacterium ulcerans* causes a chronic, necrotizing disease of the skin and the subcutaneous adipose tissue commonly known as Buruli ulcer [1]. This serious infectious disease remains a major health problem in many parts of the world, but in particular, in Western and Central Africa [2]. In spite of considerable research efforts made during the past few years transmission and environmental reservoirs of *M. ulcerans* are still incompletely characterized [1]. Endemic foci are usually linked to wetlands and riverine areas, which has lead to the assumption that *M. ulcerans* is an environmental mycobacterium and that micro-traumata of the skin may initiate infection [3]. However, isolation of the slow growing *M. ulcerans* from an environmental source has been achieved only once so far, from an aquatic insect [4]. PCR screening of environmental samples for the presence of *IS2404* has implicated insects such as biting aquatic hemiptera and mosquitoes in the transmission of *M. ulcerans* [5]–[7], but their positivity for *M. ulcerans* DNA in polymerase chain reaction (PCR) tests may be only an indicator for the presence of *M. ulcerans* or other genetically closely related mycobacteria in the environment. Although BU is known to develop in all age groups with a nearly equal gender distribution, children 15 years of age or younger make up at least 50% of all cases in Africa [8]. Occasional clustering of cases within families may reflect a common source of infection or increased genetic susceptibility to infection rather than human-to-human transmission. Seroepidemiological studies have indicated that infection with *M. ulcerans* may lead to disease only in a minority of exposed individuals [9].

Many genetic fingerprinting methods have been applied for *M. ulcerans*, including *IS2404*, *IS2606* and *IS2426* PCR [10], [11], amplified fragment length polymorphism analysis (AFLP) [12], *IS2404* restriction fragment length polymorphism analysis (RFLP) [13], [14], multi-locus sequence typing (MLST) [15]–[17], variable-number tandem repeat analysis (VNTR) [18]–[21], *IS2404-Mtb2* PCR [22], and large sequence polymorphisms [23]. Among these, AFLP [12] and VNTR typing [19], [21] were the only methods to reveal any genetic diversity among African strains.

MLST, which has now been developed for more than 50 microbial taxa [24], revealed extremely low levels of polymorphisms in several protein coding genes of African *M. ulcerans* strains [16], [17]. Analyses of the population structure of bacterial pathogens such as *M. tuberculosis* [25], *Yersinia pestis* [26] or *Salmonella enterica Typhi* [27] have shown that single nucleotide polymorphism (SNP) typing is the most suitable fine-typing method for genetically monomorphic species [24]. This prompted us to develop a SNP typing method for *M. ulcerans* strains from a BU endemic area of Ghana. We selected two Ghanaian patient isolates representing two different Ghanaian VNTR types [19] for genome re-sequencing and compared obtained sequences with the published genome sequence of the reference strain Agy99 [28]. Whole genome comparison between these three strains detected 173 SNPs in total [29], which were used for the establishment of amplification refractory mutation system (ARMS) real-time PCRs using hairpin-shaped primers [30]. Typing of 74 strains isolated from patients living in the BU endemic Densu river basin at 65 SNP loci revealed the presence of five haplotypes in addition to the Agy99 reference haplotype. Sequencing of 4 additional strains chosen on the basis of detected haplotypes enabled further differentiation of isolates. Location of the homes of patients from whom the strains were isolated facilitated a phylogeographic analysis of haplotype distribution.

## Materials and Methods

### Ethics statement

In the present study, *M. ulcerans* isolates were obtained from BU diagnostic samples. Data were analyzed anonymously and bacterial isolates delinked from the patients from whom they originated. Ethical approval to use the diagnostic specimens for immunological and microbiological research was obtained from the ethical review board of the Noguchi Memorial Institute for Medical Research, University of Ghana, Legon, Ghana. Written informed consent was provided by all patients for standard surgical treatment and anaesthesia. In addition the ethical review board requested written informed consent for taking blood samples for immunological research on a special consent form, but not for potential later investigations of bacterial isolates generated during standard diagnostic procedures.

### Mycobacterial strains and genomic DNA extraction

A total of 74 *M. ulcerans* patient isolates from a BU endemic area located in the Ga West, Ga East and Akuapim South Districts were included in the SNP typing analysis. Patients were aged 2–75 years, while 71% of the patients were younger than 15 years. In addition, *M. ulcerans* patient isolates from the Ashanti Region (Amansie West District) of Ghana and from other West-African countries (Ivory Coast, Togo, Benin, Democratic Republic of Congo and Angola) were enclosed for further SNP typing analyses (Table 1). The complete Agy99 genome sequence published in 2007 [28] and re-sequenced genomes of isolates NM20/02, NM31/04 [29], NM14/01, NM43/02, NM49/02 and NM54/02 were used as reference sequences. The genomes of strains NM14/01, NM43/02, NM49/02 and NM54/02 were analyzed at Monash University using an Illumina GAIIx Genome Analyzer. A 100x coverage per genome was obtained on average. The Short Read Mapping Package (SHRiMP) software was used for aligning the genomic reads against the target Agy99 genome. For SNP identification the Nesoni software was used. Genomic DNA was isolated by cell wall disruption and phenol-chloroform extraction as described previously [31].

**Table 1:** *M. ulcerans* strains included in the analysis.

Number of strains	Year of isolation	Place of origin	VNTR type^1^
1 (Agy99)	1999	Ga District, Ghana	BD/BAA
38 (NM20/02)	2002	Ga District, Ghana	BD/B
21	2003	Ga District, Ghana	BD/B
5	2004	Ga District, Ghana	n.t.^2^
8	2005	Ga District, Ghana	n.t.
1	2006	Ga District, Ghana	n.t.
1	2007	Ga District, Ghana	n.t.
3 (NM31/04)	2004	AW District, Ghana	C/BAA
7	1997–2001	Benin	n.t.
3	n.p.^3^	DRC	n.t.
1	1997	Togo	n.t.
2	1994	Ivory Coast	n.t.
2	1996	Angola	n.t.

1 ST1/MIRU1 allele [19].

2 not tested.

3 not provided.

### SNP typing

Real-time PCR hairpin primer (HP) assays [30] were used to detect SNPs in the 74 Ghanaian *M. ulcerans* strains. Real-time PCR was performed using Power SYBR green 1x PCR Master Mix (Applied Biosystems), 5 ng genomic DNA and 0.3 µM forward and reverse primers each in a total volume of 25 µl. Reactions were carried out in a Step One Plus Real-time PCR system (Applied Biosystems) with a 96-well block. Thermal conditions were as follows and as described previously [30]: stage 1, 95°C for 10 min, 70°C for 30 s; stage 2, 72°C for 30 s, 95°C for 20 s, 69°C for 30 s, lowering one degree in the last step for every cycle during 10 cycles; stage 3, 72°C for 30 s, 95°C for 20 s, and 60°C for 30 s, repeated 40 times; Melt curve stage, 95°C for 15 s, 60°C for 1 min, 95°C for 15 s. Data were collected in the last step of stage 3 and after the Melt curve stage for analysis with the Step One Software version 2.0 (Applied Biosystems). SNPs were detected by ARMS assays and for each assay two PCRs with two sets of PCR primers were performed in parallel. Each PCR reaction contains SNP-specific primers, which are designed to be either fully complementary to the DNA template or mismatched at the 3′end nucleotide. As reactions with totally complementary primers have a more rapid developing fluorescence curve and an earlier cycle-threshold, differences between the two reactions allow the detection of SNPs.

The hairpin-shaped primers were designed as described previously [30]. In the first step linear primers were designed with Primer3 [32] to produce short amplicons (30 to 90 bp) and to anneal between 60 and 65°C. A tail was added to the 5′ end of the SNP-detecting primer in order to produce a stem with the 3′ end of the primer. The stem was designed with mfold software (http://www.bioinfo.rpi.edu/applications/mfold/old/dna/) to have a melting temperature of 67 to 70°C with a free energy of between −0.5 and −2.0. Primers are provided in Table S1.

### Validation of SNP typing assays

Assays were validated on the published genome Agy99 as well as the reference strains NM20/02, NM31/04, NM14/01, NM43/02, NM49/02 and NM54/02 to confirm the presence of each allele and to verify the performance of SNP assays. Assays with genomic DNA samples from clinical isolates were considered reliable only if the cycle thresholds generated in the paired wells differed by three or more cycles and if the melting curves of paired wells were coherent. ARMS assays with low discriminatory power were not included into the standard set of 65 SNP typing assays. Sanger DNA sequencing of PCR products was used to validate a selected subset of SNPs. Primers used for PCR and sequencing were designed using Primer3 [32] software. PCR was performed using FirePol 10x buffer and 0.5 µl FirePolTaq-Polymerase (Solis BioDyne), 5 ng genomic DNA, 0.72 µM forward and reverse primers each, 2 mM MgCl_2_ and 0.4 mM dNTPs (Sigma) in a total volume of 25 µl. PCR reactions were carried out in a Gene Amp PCR System 9700 PCR machine (Applied Biosystems). Thermal conditions for PCR amplification of *M. ulcerans* genomic DNA were as follows: initial denaturation step, 94°C for 5 min; 32 cycles: 94°C for 30 s (denaturation), 60°C for 30 s (annealing), 72°C for 1 min (elongation); final extension step, 72°C for 10 min. PCR products were analyzed on 1% agarose gels. PCR products were purified using the Nucleo Spin Extract II Kit (Macherey-Nagel). Sequencing of purified PCR products was done by Macrogen (World Meridian Venture Center, Seoul/Korea). Sequencing was conducted by the Sanger method using BigDye™ terminator cycling conditions using the Automatic Sequencer 3730xl (Applied Biosystems).

Several random SNP loci were validated by Sanger sequencing in order to verify the ARMS approach for a differentiation of *M. ulcerans* isolates. Additionally, validation of pivotal SNPs loci revealing different haplotypes within the endemic area around the Ga District as well as differences between Amansie West District strains and isolates from other African countries was carried out. 100% of 36 randomly chosen SNP loci tested in reference strains Agy99, Nm20/02 and Nm31/04 were reconfirmed by Sanger sequencing. 100% of 70 significant SNP loci dividing Ga District strains into 5 haplotypes other than Agy99 and distinguishing Amansie West District as well as African strains were likewise verified by Sanger sequencing. In contrast, four real-time PCR typing results, which were validated because of unique allele occurrences in certain Ga District isolates diverged from reconfirmatory Sanger sequencing analysis. Subsequent repetition of real-time PCRs in duplicates revised the initial real-time PCR analyses and confirmed Sanger sequencing results.

### Phylogenetic analysis

MEGA software version 4.1 (beta) [33] was used to reconstruct the neighbor-joining tree based on SNP typing data (Phylogeny Test and options: Bootstrap 1000 replicates; Gaps/Missing Data: Complete Deletion; Codon Positions: 1st+2nd+3rd+Noncoding; Model: Nucleotide, Number of differences; Substitutions to include: Transitions + Transversions; Pattern among lineages: Same  =  Homogeneous; Rates among sites: Uniform rates).

We created a map of West Africa by using the map creator tool of Epi Info version 3.5.1 in order to illustrate detected SNP patterns in different African countries.

## Results

### Development of ARMS SNP typing assays

**Figure 1:**
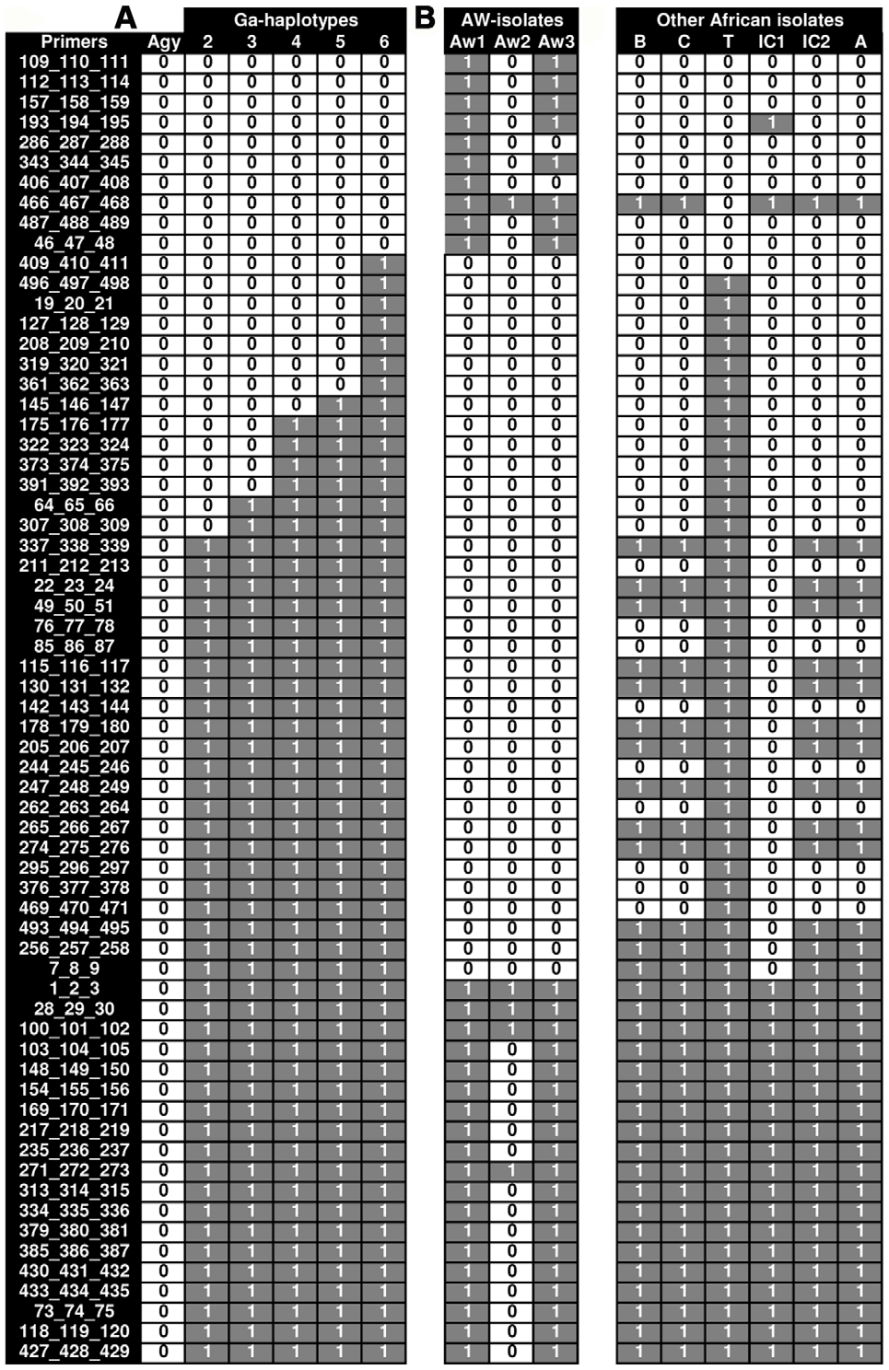
SNP typing analysis of African *M. ulcerans* isolates. **A** *M. ulcerans* isolates from the Densu river basin of Ghana were analyzed at 65 SNP loci (Primer IDs) by real-time PCRs. Base exchanges relative to the reference sequence of strain Agy99 were registered as 1 (grey). Allele matches with Agy99 were recorded as 0 (white). 5 haplotypes in addition to haplotype 1 (Agy99) could be distinguished on the basis of 14 SNP loci. **B** SNP typing results of strains from a second BU endemic area of Ghana as well as from additional African countries carried out with the set of SNP assays developed by whole genome sequencing of Ghanaian isolates. AW: Amansie West; Ga: strains from the Densu river basin; IC: Ivory Coast; T: Togo; B: Benin; C: Democratic Republic of Congo; A: Angola.

Recently we have compared 454 and Illumina genome sequencing data of *M. ulcerans* patient isolates NM20/02 and NM31/04 originating from two different BU endemic areas of Ghana with the published genome sequence of the Ghanaian strain Agy99 [29]. Based on the identification of 173 SNPs we have developed medium-throughput ARMS-based real-time PCR SNP assays with hairpin-shaped (HP) primers. Initial ARMS assays were successful for 108 of 173 detected SNP loci at predefined optimal conditions. We were able to discriminate alleles under a single standard condition at 73/108 (67.6%) SNP loci. This is close to the success rate of 72.4% reported by Hazbon and Alland for their first round of HP-assay design [30]. Real-time PCR analysis revealed sequencing errors in the Agy99 reference sequence at eight of these loci, which were thus not suitable for further typing analyses. Redesign of the 100 initially failed assays and ongoing whole-genome sequencing of additional isolates will increase the pool of discriminatory SNP assays for future analyses of the population structure of African *M. ulcerans*. Developed SNP assays were used to type a collection of strains from two different BU endemic areas of Ghana and other African countries (Figure 1).

### Different clonal complexes of *M. ulcerans* dominate in the BU endemic regions of Africa

**Figure 2:**
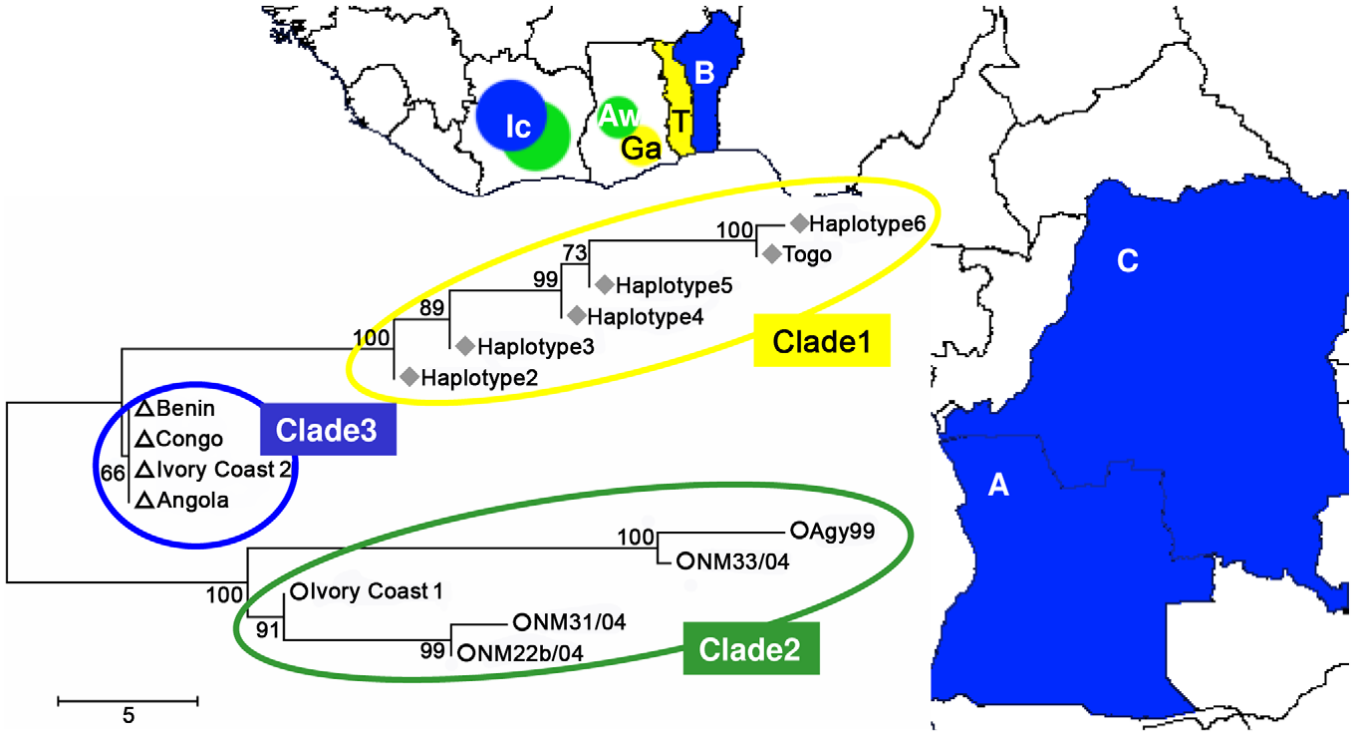
Geographical distribution of African *M. ulcerans* clades. Map of West-Africa, showing the distribution and SNP haplotypes of three African *M. ulcerans* clades. Clade 1: yellow; clade 2: green; clade 3: blue. AW: Amansie West; Ga: strains from the Densu river basin; IC: Ivory Coast; T: Togo; B: Benin; C: Democratic Republic of Congo; A: Angola. A neighbor-joining tree shows sub-grouping of detected haplotypes from the Densu river basin together with the only strain from Togo into clade 1, strains from AW together with strain Agy99 and strain 1 from the Ivory Coast into clade 2 and all other strains from additional African countries into clade 3 (scale: number of differences at the SNP loci tested).

SNP typing of three strains from the Amansie West District of Ghana including the sequenced Amansie West reference strain NM31/04 revealed differences at 24 of the 65 SNP loci analyzed (37%) between these isolates (Figure 1B). In comparison to Agy99 differences at 29, 27 and 5 loci were found. A neighbor-joining tree analysis sub-grouped haplotypes from the two different BU endemic areas of Ghana into two clades (Figure 2). Clade 1 comprises strains isolated between 2001 and 2007 in the Densu river basin, which could be differentiated into 6 haplotypes (Figure 1A). All Amansie West District isolates are sub-grouped together with the 1999 isolate Agy99 into clade 2 (Figure 2).

Typing of patient isolates from other BU endemic African countries at the 65 SNP loci yielded 2 of 15 strains with patterns similar to haplotypes found in Ghana (Figure 1A and B). The only strain available from Togo had a haplotype which differed at only one locus from haplotype 6 found in the Densu river basin. One of the two analyzed strains from the Ivory Coast had a haplotype similar to the haplotypes of strains from the Amansie West District of Ghana. The other isolate from the Ivory Coast as well as all seven analyzed strains from Benin, three strains from the Democratic Republic of Congo (DRC) and two strains from Angola shared a distinct SNP pattern when compared to the Ghanaian isolates (Figure 1A and B). Thus, SNP typing results of clinical isolates from Ghana and isolates from other African countries revealed a neighbor-joining tree with 3 main branches. The strain from Togo is sub-grouped together with haplotypes 2–6 into clade 1, while strain 1 from the Ivory Coast is classed with Amansie West District isolates and Agy99 into clade 2. The other 13 West-African strains are sub-grouped into clade 3 (Figure 2).

### SNP typing of *M. ulcerans* patient isolates from a BU endemic area of Ghana identifies ten haplotypes belonging to a dominating clonal complex

Using the 65 established ARMS assays we SNP-typed 74 *M. ulcerans* patient isolates collected between 2001 and 2007 from the BU endemic Densu river basin of Ghana, from which the sequenced strains Agy99 and NM20/02 originated. Within this group of 74 strains of common geographical origin, differences at 14 of the 65 SNP loci tested (22%) were observed (Figure 1A). Altogether five haplotypes (designated haplotypes 2–6) other than the Agy99 associated haplotype 1 could be distinguished. Haplotypes 2–6 differed at 41, 43, 47, 48 and 55 of the 65 analyzed SNP loci from haplotype 1 (strain Agy99), respectively.

**Figure 3:**
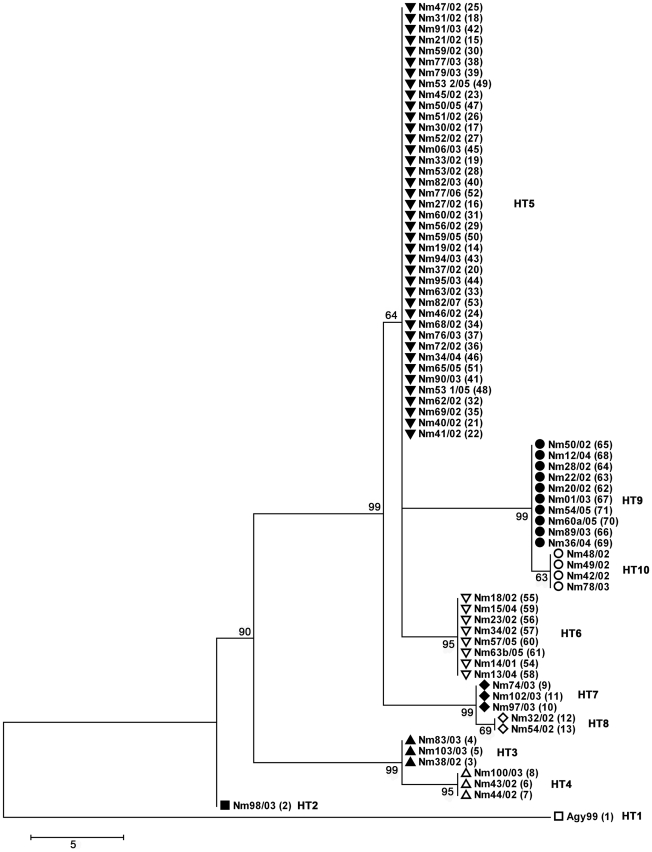
Neighbor-joining tree of 75 Ghanaian *M. ulcerans* isolates. 75 *M. ulcerans* isolates were aligned based on their SNP type (scale: number of differences at the SNP loci tested). HT  =  haplotype.

Based on detected haplotypes we re-sequenced 4 representative strains NM14/01 (haplotype 5), NM43/02 (haplotype 3), NM49/02 (haplotype 6) and NM54/02 (haplotype 4) in order to further differentiate strains from the same haplotype. We detected new unique SNPs in NM43/02 (26 SNPs), NM54/02 (11 SNPs), NM14/01 (9 SNPs) and NM49/02 (4 SNPs) and established 24 new assays, which enabled a segregation of each haplotype into two haplotypes (Figure S1). A phylogenetic tree for haplotypes 1–10 is shown in Figure 3.

### Temporal and spatial distribution of haplotypes

In contrast to the generally rarer haplotypes 2–4; 6–8 and 10, haplotypes 5 and 9 were found within each time interval (one year) from 06/2001 to 06/2006 (data not shown). Haplotypes 1 and 2 were not found again in the whole strain collection. Possible explanations include the actual absence of these haplotypes from the residual clonal *M. ulcerans* complex in the Densu river basin as well as phylogenetic or sampling bias.

**Figure 4:**
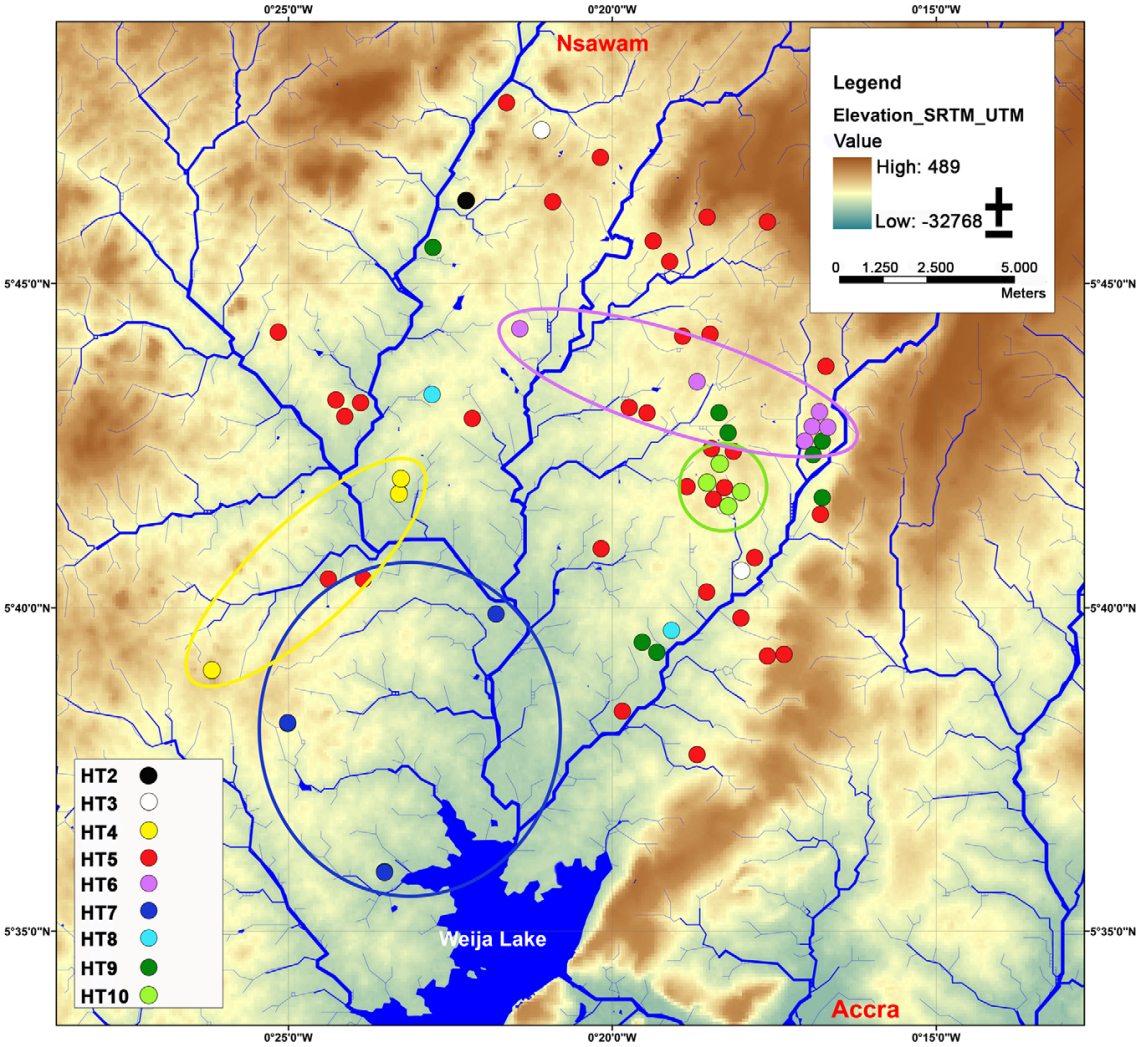
Geographical distribution of *M. ulcerans* haplotypes. Map of the Densu river basin, showing the homes of patients from whom the strains have been isolated between 2001 and 2006 (colored dots). Haplotypes 2 (black), 3 (white), 4 (yellow), 6 (purple), 7 (dark blue), 8 (light blue), 9 (dark green), 10 (light green) are unevenly distributed, whereas haplotype 5 (red) co-localizes with all other haplotypes. The background map was created using elevation data from the Shuttle Radar Topography Mission (SRTM). Water bodies were classified using optical data from Landsat ETM and radar data from TerraSAR-X.

For a phylogeographic analysis the homes of patients from whom the strains were isolated were marked in a map, depicting the distribution of haplotypes (Figure 4). Haplotypes 4, 6, 7, 9 and 10 appear to be unevenly distributed; i.e. they were found only in certain parts of the BU endemic area. Haplotype 10 was even found only within one small village. In contrast, the most prevalent haplotype (haplotype 5) co-localized with all other haplotypes. Interestingly, two *M. ulcerans* isolates of identical haplotype (haplotype 4) were isolated from two patients coming from the same household, suggesting a common source of infection.

## Discussion

Previous investigations on the genetic diversity of *M. ulcerans* by comparative genomic hybridization analysis enabled differentiation of a world-wide collection of strains into two main lineages and six continental haplotypes [34]. However, phylogeographic and transmission pathway analysis requires high-resolution fine typing of strains from the same BU endemic region. This has been accomplished so far only for a few *M. ulcerans* populations and at low resolution. VNTR analysis of a Ghanaian strain collection based on the polymorphic loci ST1 and MIRU1 revealed the presence of three distinct allele combinations (BD/B, C/BAA and BD/BAA) in Ghana. All isolates from the Buruli ulcer endemic Densu river basin tested, except for Agy99, displayed combination BD/B [19]. Our recent genome re-sequencing analysis [29] compared the two Ghanaian VNTR type reference strains NM20/02 (BD/B) and NM31/04 (C/BAA) to Agy99 (BD/BAA) with the goal of detecting single nucleotide polymorphisms suitable for development of a fine-typing method. By selection of isolates with the three prevalent VNTR types in Ghana we expected to capture as much of the genetic variation present in the Ghanaian *M. ulcerans* population as possible. Our assumption was reinforced by re-sequencing four *M. ulcerans* strains initially grouped into haplotypes 3–6. Only 16 additional SNPs could be detected by comparison of the four strains to reference strain Agy99, while 50 unique SNPs could be identified by comparing the four strains among themselves. We anticipated that detected SNPs will provide the first useful genetic markers for phylogeographic and transmission pathway analyses at least in the Densu river basin and other BU endemic areas of Ghana.

We have identified 10 different haplotypes in a relatively small BU endemic area within the Densu river basin. Haplotypes 1–10 are descendants of a founder haplotype that has spread over the district; the most common haplotype 5 may represent this founder haplotype. Analysis of this spatial distribution of haplotypes indicates that emerging new haplotypes do not readily spread over the entire endemic area, but form focal transmission clusters.

Our data are comparable to studies of other genetically monomorphic organisms like *Salmonella typhi*, which report multiple strain types circulating within a specific location [35]–[37]. Comparison of typing results in strains from two geographically separate BU endemic areas in the Densu river basin and the Amansie West Districts of Ghana uncovered a total of 61 differing alleles at 65 SNP loci. Within each of the two endemic areas SNP variation was significantly smaller (14 and 24 differences, respectively) than the overall variation between the two endemic areas. These results indicate the dominance of two different clonal complexes in the two separate Ghanaian BU endemic areas. SNP typing of two strains from Ghanaian neighboring countries showed similar SNP patterns when compared to AW District isolates (strain 1 from the Ivory Coast) or Ga District haplotypes (strain from Togo). Typing of 13 strains collected in additional African countries (Benin, DRC, Angola, strain 2 from the Ivory Coast) revealed a completely new SNP pattern compared to all other isolates. On the basis of typing with the Ghanaian set of SNPs these 13 strains could not be distinguished among each other and were thus clustered together into a clade. This clustering may however represent a phylogenetic discovery bias, i.e grouping of actually diverse strains leading to a so called “branch collapse”. Future addition of SNP loci identified by genome re-sequencing of a comprehensive pan-African selection of *M. ulcerans* isolates will lead to a further subdivision and differentiation of African strains. Phylogenetic discovery bias is implicit to SNP typing and will continue to exist as long as not every single sample will be sequenced. It will become smaller though with every additional re-sequenced genome. SNP typing based on a wider range of SNPs may therefore yield evidence for genetic divergence of strains from Benin, DRC, and strain two from the Ivory Coast. Hence, phylogeographic analyses in other African BU endemic areas will require whole genome comparison of strains from that area to develop a local set of informative SNPs. This is supported by our recent identification of seven insertion sequence element-related SNP types within Africa [38].

*M. ulcerans* has evolved from the aquatic environmental *M. marinum* and seems to have adapted to a more stable ecological niche [28]. Gain of the immunosuppressive toxin mycolactone is accompanied by loss of highly immunogenic proteins [39], suggesting an adaptation to survival in host environments that are screened by immunological defense mechanisms. Serological analyses have indicated that many individuals living in BU endemic areas are exposed to *M. ulcerans,* whereas relatively few develop clinical disease [9]. There is no published evidence for direct person to person transmission of *M. ulcerans*. Based on numerous reports demonstrating an association of BU with slow flowing or stagnant water bodies, it is therefore commonly assumed that infection takes place through trauma of the skin or insect bites via an environmental reservoir in the aquatic ecosystems. Both biotic components, such as biofilms and aquatic invertebrate species are being considered as potential vectors and/or reservoirs [5], [6], [40]–[42]. Recent findings in south-eastern Australia have implicated mammals as environmental reservoir (Fyfe et al., submitted) and mosquitoes as vectors of *M. ulcerans* [43], [44]. While large numbers of possums in a BU-endemic area of Australia are infected with *M. ulcerans*, a similar mammalian reservoir has not been identified in BU endemic regions of Africa. If such a reservoir plays a role in transmission, spread of *M. ulcerans* from chronic, ulcerated lesions to insect vectors or another currently unknown environmental reservoir should be considered. Subsequent infection of individuals living in the same settlements may be responsible for the focal transmission patterns, such as of haplotype 10, which was found only within one small village.

The Densu river basin represents one of the coastal drainage systems of Ghana. Its water is collected in the Weija Lake, which developed after construction of a dam at the river's mouth in the 1970s. If *M. ulcerans* bacteria would be carried freely by the flow of water of the Densu river and its tributaries, haplotypes present in upper parts of the river system should be represented in the lower parts. However, among patient isolates coming from the lower part of the basin close to the Weija Lake the rare haplotypes 4 and 7 dominated. *M. ulcerans* bacteria that are swept downstream in the river water thus do not seem to play a major role in transmission.

Results of this retrospective pilot study indicate that future longitudinal micro-epidemiological studies involving SNP typing of isolates may give deeper insight into transmission pathways and relevant reservoirs of *M. ulcerans.*

## Supporting Information

Figure S1 SNP typing analysis of *M. ulcerans* isolates from the Ga district of Ghana. *M. ulcerans* isolates from the Densu river basin of Ghana were analyzed at 89 SNP loci (Primer IDs) by real-time PCRs. SNPs were registered as 1 (grey). Agy = 1; Nm98/03 = 2; Nm38/02 = 3; Nm83/03 = 4; Nm103/03 = 5; Nm43/02 = 6; Nm44/02 = 7; Nm100/03 = 8; Nm74/03 = 9; Nm97/03 = 10; Nm102/03 = 11; Nm32/02 = 12; Nm54/02 = 13; Nm19/02 = 14; Nm21/02 = 15; Nm27/02 = 16; Nm30/02 = 17; Nm31/02 = 18; Nm33/02 = 19; Nm37/02 = 20; Nm40/02 = 21; Nm41/02 = 22; Nm45/02 = 23; Nm46/02 = 24; Nm47/02 = 25; Nm51/02 = 26; Nm52/02 = 27; Nm53/02 = 28; Nm56/02 = 29; Nm59/02 = 30; Nm60/02 = 31; Nm62/02 = 32; Nm63/02 = 33; Nm68/02 = 34; Nm69/02 = 35; Nm72/02 = 36; Nm76/03 = 37; Nm77/03 = 38; Nm79/03 = 39; Nm82/03 = 40; Nm90/03 = 41; Nm91/03 = 42; Nm94/03 = 43; Nm95/03 = 44; Nm06/03 = 45; Nm34/04 = 46; Nm50/05 = 47; Nm53_1/05 = 48; Nm53_2/05 = 49; Nm59/05 = 50; Nm65/05 = 51; Nm77/06 = 52; Nm82/07 = 53; Nm14/01 = 54; Nm18/02 = 55; Nm23/02 = 56; Nm34/02 = 57; Nm13/04 = 58; Nm15/04 = 59; Nm57/05 = 60; Nm63b/05 = 61; Nm20/02 = 62; Nm22/02 = 63; Nm28/02 = 64; Nm50/02 = 65; Nm89/03 = 66; Nm01/03 = 67; Nm12/04 = 68; Nm36/04 = 69; Nm60a/05 = 70; Nm54/05 = 71; Nm42/02 = 72; Nm48/02 = 73; Nm49/02 = 74; Nm78/03 = 75.
(2.97 MB TIF)

Table S1 Real-time PCR primers. Primers and hairpin primers used for real-time PCR amplification refractory mutation assays.
(0.28 MB DOC)
