# Supplementary figures and images for: Single Nucleotide Polymorphism Typing of Mycobacterium ulcerans Reveals Focal Transmission of Buruli Ulcer in a Highly Endemic Region of Ghana
Source: PLoS Negl Trop Dis. 2010 Jul 20;4(7):e751. doi: 10.1371/journal.pntd.0000751 (PMC2907412; doi:10.1371/journal.pntd.0000751)

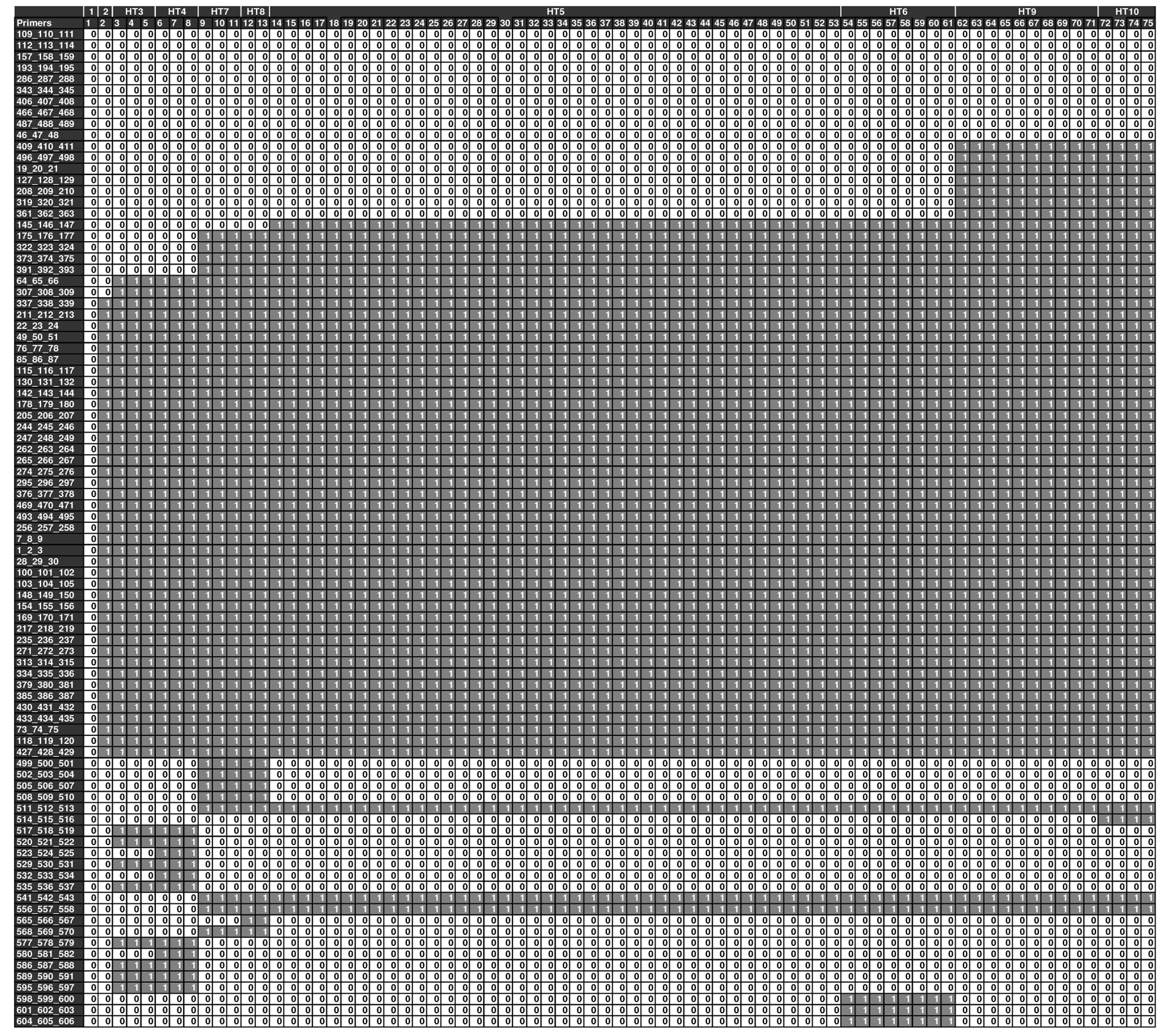

Supplement: Figure S1 — SNP typing analysis of M. ulcerans isolates from the Ga district of Ghana. M. ulcerans isolates from the Densu river basin of Ghana were analyzed at 89 SNP loci (Primer IDs) by real-time PCRs. SNPs were registered as 1 (grey). Agy = 1; Nm98/03 = 2; Nm38/02 = 3; Nm83/03 = 4; Nm103/03 = 5; Nm43/02 = 6; Nm44/02 = 7; Nm100/03 = 8; Nm74/03 = 9; Nm97/03 = 10; Nm102/03 = 11; Nm32/02 = 12; Nm54/02 = 13; Nm19/02 = 14; Nm21/02 = 15; Nm27/02 = 16; Nm30/02 = 17; Nm31/02 = 18; Nm33/02 = 19; Nm37/02 = 20; Nm40/02 = 21; Nm41/02 = 22; Nm45/02 = 23; Nm46/02 = 24; Nm47/02 = 25; Nm51/02 = 26; Nm52/02 = 27; Nm53/02 = 28; Nm56/02 = 29; Nm59/02 = 30; Nm60/02 = 31; Nm62/02 = 32; Nm63/02 = 33; Nm68/02 = 34; Nm69/02 = 35; Nm72/02 = 36; Nm76/03 = 37; Nm77/03 = 38; Nm79/03 = 39; Nm82/03 = 40; Nm90/03 = 41; Nm91/03 = 42; Nm94/03 = 43; Nm95/03 = 44; Nm06/03 = 45; Nm34/04 = 46; Nm50/05 = 47; Nm53_1/05 = 48; Nm53_2/05 = 49; Nm59/05 = 50; Nm65/05 = 51; Nm77/06 = 52; Nm82/07 = 53; Nm14/01 = 54; Nm18/02 = 55; Nm23/02 = 56; Nm34/02 = 57; Nm13/04 = 58; Nm15/04 = 59; Nm57/05 = 60; Nm63b/05 = 61; Nm20/02 = 62; Nm22/02 = 63; Nm28/02 = 64; Nm50/02 = 65; Nm89/03 = 66; Nm01/03 = 67; Nm12/04 = 68; Nm36/04 = 69; Nm60a/05 = 70; Nm54/05 = 71; Nm42/02 = 72; Nm48/02 = 73; Nm49/02 = 74; Nm78/03 = 75. (2.97 MB TIF) [file pntd.0000751.s001.tif]
