# Supplementary material for: Single Nucleotide Polymorphism Typing of Mycobacterium ulcerans Reveals Focal Transmission of Buruli Ulcer in a Highly Endemic Region of Ghana
Source: PLoS Negl Trop Dis. 2010 Jul 20;4(7):e751. doi: 10.1371/journal.pntd.0000751 (PMC2907412; doi:10.1371/journal.pntd.0000751)
Supplement: Table S1 — Real-time PCR primers. Primers and hairpin primers used for real-time PCR amplification refractory mutation assays. (0.28 MB DOC) [file pntd.0000751.s002.doc]

**Table S1. Real-time PCR primers.**

| **Genome pos.** | **Real-time PCR ID** | **Primer sequence** |
| --- | --- | --- |
|  |  |  |
| **1329454** | 109 | aggcaaaCCGCTTGATGTTTGCCT |
|  | 110 | cggcaaagGCTTGATGTTTGCCG |
|  | 111 | CAGGACGAGACAGCGGAC |
| **1596932** | 112 | tcacccATGGCGCACTGGGTGA |
|  | 113 | ccacccGcCGCACTGGGTGG |
|  | 114 | CCTGGAATCCATCCCGA |
| **2602472** | 157 | ggtactggggCACTCGTAGTAGCCCAGTACC |
|  | 158 | tgtactggggCACTCGTAGTAGCCCAGTACA |
|  | 159 | CGATCTTGAGGACGACGAC |
| **3159571** | 193 | tcagtccAGCCGCCGGACTGA |
|  | 194 | ccagtccAGCCGCCGGACTGG |
|  | 195 | GCCTTCGAACACGGTGAC |
| **5070687** | 286 | aagccaCCGCTAGGCTTGGCTT |
|  | 287 | gagccaCCGCTAGGCTTGGCTC |
|  | 288 | CTTCAACAAATGACCCGGAC |
| **1009532** | 343 | gcatcccACCACCTCGGGGATGC |
|  | 344 | acatcccACCACCTCGGGGATGT |
|  | 345 | ATCGTGAACCTCGAAGCAAT |
| **2974547** | 406 | gcgtagaggTCGACCCGCTCTACGC |
|  | 407 | acgtagagccAACTCGACCCGCTCTACGT |
|  | 408 | GCACGTGGTCATACAGATCG |
| **4371152** | 466 | ggccaagCCTCATTTCGATACTTGGCC |
|  | 467 | cgccaagCCTCATTTCGATACTTGGCG |
|  | 468 | CGACTAGCTCGGCGGAC |
| **5346222** | 487 | cgggtcTGCACTCTGGTGACCCG |
|  | 488 | agggtcacTTTGCACTCTGGTGACCCT |
|  | 489 | AACGATGTACCTTTGACCGC |
| **446532** | 46 | ttcggctGGCTGATCGAGCCGAA |
|  | 47 | ctcggctGGCTGATCGAGCCGAG |
|  | 48 | GATGATGCGGCTTTCCAG |
| **3042792** | 409 | ccagCCAGGACCAGGGCTGG |
|  | 410 | tcagccACCAGGACCAGGGCTGA |
|  | 411 | GTTGTGGCACACCCGAC |
| **5603344** | 496 | tgatcggaCGGCGAGAATCCGATCA |
|  | 497 | ggatcggCGGCGAGAATCCGATCC |
|  | 498 | GCTGAGCCGCTTGCAC |
| **4321063** | 19 | CTGCCGCGTATTTACCACCGGCAG |
|  | 20 | TTGCCGCGTATTTACCACCGGCAA |
|  | 21 | CTCACGGCATGATGAGTGTT |
| **2123161** | 127 | cgttccccTGCGGGTGGGAACG |
|  | 128 | ggttcccTGCGGGTGGGAACC |
|  | 129 | CGGAACCCCAAGAGCTG |
| **3545128** | 208 | gacagCgCCCTCGGGCTGTC |
|  | 209 | aacagCgCCCTCGGGCTGTT |
|  | 210 | CAAGGGCAGCAGAGATTCC |
| **358130** | 319 | gaccgCCCGCCGACGGTC |
|  | 320 | aaccgtTCCCGCCGACGGTT |
|  | 321 | GTGGCGGTGTTCGAGAAG |
| **1744600** | 361 | ccaccgCAACCCGTCCGGTGG |
|  | 362 | gcaccgCAACCCGTCCGGTGC |
|  | 363 | GTTCAACTCGCAGACTTGGG |
| **2455125** | 145 | aacctgtgAGCCGCTCCCACAGGTT |
|  | 146 | gacctGCCGCTCCCACAGGTC |
|  | 147 | CAGATCCTTCTCGGTGACG |
| **2827516** | 175 | aaaatgacggGCGGATGCGTCATTTT |
|  | 176 | gaaatgacggGCGGATGCGTCATTTC |
|  | 177 | CGGTGGATAGTAGTCGGACG |
| **376413** | 322 | ccggatcGCGACCAGGATCCGG |
|  | 323 | tcggatcGGCGACCAGGATCCGA |
|  | 324 | ACAGTCGGTGAGATGGTCTG |
| **1953705** | 373 | cgacctgACCGAAGTGGCAGGTCG |
|  | 374 | tgacctACCGAAGTGGCAGGTCA |
|  | 375 | AGATACAGGCCGCAATCATC |
| **2403617** | 391 | cggctatcTcACCTCCGGATAGCCG |
|  | 392 | tggctatccTTGACCTCCGGATAGCCA |
|  | 393 | GAGGTCTGGCGAACAACTTC |
| **643120** | 64 | cgggcTTCGCACCAGTAGCCCG |
|  | 65 | agggctCTTCGCACCAGTAGCCCT |
|  | 66 | CAGCGCGTGGTCCTCTAC |
| **5580480** | 307 | tgcggTGGATGTTGGCCGCA |
|  | 308 | agcgGTGGATGTTGGCCGCT |
|  | 309 | AAGAAGGTCGTCATCATCGG |
| **554471** | 337 | tgcgGTAACCCGAGCCGCA |
|  | 338 | cgcggTCGTAACCCGAGCCGCG |
|  | 339 | TACAGCGACTACATGGTGCC |
| **3589134** | 211 | atcagcttgGACAGAATTTGGCAAGCTGAT |
|  | 212 | gtcagcttgGACAGAATTTGGCAAGCTGAC |
|  | 213 | TACAGCGATCCAGGTCATCA |
| **4631398** | 22 | GAAAGCCAGAATCTGGCGGGCTTTC |
|  | 23 | AAAAGCCAGAATCTGGCGGGCTTTT |
|  | 24 | GAGGTGGTTCGCGATCTG |
| **453370** | 49 | accgttgCgCGAAGAGGTCAACGGT |
|  | 50 | gccgttgCCGAAGAGGTCAACGGC |
|  | 51 | ACCACTTCTTGCGCACTTCT |
| **759634** | 76 | cggaaaccGGCGGTGGGTTTCCG |
|  | 77 | tggaaaccGGCGGTGGGTTTCCA |
|  | 78 | AACCAGGTTCGAGTGGCTC |
| **886275** | 85 | ggtacgcTCTGCGCTCGCGTACC |
|  | 86 | agtacgcATCTGCGCTCGCGTACT |
|  | 87 | AGCCCGGACCTAGTCGATAC |
| **1699678** | 115 | tggttttcgTTCGATTGTAAGTCGAAAACCA |
|  | 116 | gggttttcgTTCGATTGTAAGTCGAAAACCC |
|  | 117 | GTGTCCATGCGATTCAAGAC |
| **2283072** | 130 | ggcaggCGCGGACTCCTGCC |
|  | 131 | agcaggaCCGCGGACTCCTGCT |
|  | 132 | TCACGTTCATCCTTGCTTTG |
| **2433594** | 142 | atgccccCCCGTCTCGGGCAT |
|  | 143 | gtgccCCCGTCTCGGGCAC |
|  | 144 | AAGACGTCATACTCGGCCTG |
| **2829304** | 178 | agggCGGCTGCATGCCCT |
|  | 179 | cgggccGCTGCATGCCCG |
|  | 180 | CTTCACAACGGTGACACCAC |
| **3497043** | 205 | tacgcagCGCTGCCTCCTGCGTA |
|  | 206 | gacGCTGCCTCCTGCGTC |
|  | 207 | CAGGTAGTCCCCCAGCCT |
| **4280152** | 244 | aagcgcTTCCGCAGTTGCGCTT |
|  | 245 | gagcgTCCGCAGTTGCGCTC |
|  | 246 | ATCGATTCTCGGATCAGGTC |
| **4339801** | 247 | ggaaattcgGATGATAGGGCCGAATTTCC |
|  | 248 | agaaattcGGATGATAGGGCCGAATTTCT |
|  | 249 | AATATCGATGAGTTTCCCGC |
| **4513198** | 262 | ggcgaaaagGGTGTCGTTTTCGCC |
|  | 263 | agcgaaaaccCGGTGTCGTTTTCGCT |
|  | 264 | AGACCCCGGTTTTCCAGTT |
| **4646293** | 265 | agcaccaGCCCGTACTCGGTGCT |
|  | 266 | ggcacccCCCGTACTCGGTGCC |
|  | 267 | AACGGAACCCCAGGTTTTAC |
| **4981352** | 274 | acgggCACGTCGGAAACCCGT |
|  | 275 | gcgggACGTCGGAAACCCGC |
|  | 276 | GACGTCGGTTACGAGTCCC |
| **5321490** | 295 | tggaactGCCTTGGCTACCAGTTCCA |
|  | 296 | cggaactgCCTTGGCTACCAGTTCCG |
|  | 297 | AGAACTCATCCAAACGCGAG |
| **1958865** | 376 | agcgcACAGCGTGTCGCGCT |
|  | 377 | ggcgAgAGCGTGTCGCGCC |
|  | 378 | AGGTCAACGAAATCGGCTAC |
| **4434770** | 469 | gcgcgcCGATGCTTACGTCGCGC |
|  | 470 | ccgcgCcATGCTTACGTCGCGG |
|  | 471 | CGCGCTATTACGTCAGCATA |
| **5593101** | 493 | gccggACTCTCCCCCACCGGC |
|  | 494 | accggtACTCTCCCCCACCGGT |
|  | 495 | GGTCAGGTCGAGGACCAC |
| **4449315** | 256 | ggcagtGTTGCTGCTCTCACTGCC |
|  | 257 | agcagtgaCGTTGCTGCTCTCACTGCT |
|  | 258 | GCAGGTCGGGGTAATCG |
| **1321620** | 7 | GCAGGATTCTCAGGCGTTGAATCCTGC |
|  | 8 | CCAGGATTCTCAGGCGTTGAATCCTGG |
|  | 9 | AAATCCGACTCCAACAGCAT |
| **32013** | 1 | AGACTTCACAGCTGCTCGGTGAAGTCT |
|  | 2 | GGACTTCACAGCTGCTCGGTGAAGTCC |
|  | 3 | GCCACTATCAGTCCCTGGAG |
| **5388861** | 28 | AGATTCGTTGAGCTGTTGGACAACGAATCT |
|  | 29 | GGATTCGTTGAGCTGTTGGACAACGAATCC |
|  | 30 | GTGACGTAGGCCTTCACCC |
| **1183060** | 100 | aggacaaCGCCGAGTTGTTGTCCT |
|  | 101 | gggacaaCGCCGAGTTGTTGTCCC |
|  | 102 | GACTCCAGACGAACCCAGC |
| **1260409** | 103 | ataacgcagCAAACACCCAGTGCGTTAT |
|  | 104 | gtaacgcagCAAACACCCAGTGCGTTAC |
|  | 105 | GAATAGCATGTGCCGGGTAT |
| **2500819** | 148 | cagcatgaCGCCGCCTCATGCTG |
|  | 149 | gagcatgCGCCGCCTCATGCTC |
|  | 150 | CCAAGACCAGTGCCGACTAT |
| **2507419** | 154 | ttgtggggGGACAGGGCCCACAA |
|  | 155 | ctgtggggGGACAGGGCCCACAG |
|  | 156 | GCCTGGTGTGCTATGTTTCC |
| **2737448** | 169 | gaaccgcTTCGTGTTCCtGCGGTTC |
|  | 170 | aaaccgcTTCGTGTTCCtGCGGTTT |
|  | 171 | CTTCAGAGCCTACTCGCCC |
| **3621904** | 217 | tgttgggCTGCGCCCCCAACA |
|  | 218 | cgttgggCTGCGCCCCCAACG |
|  | 219 | AGTTTTTGTCCATTGGGCTG |
| **3910322** | 235 | ctcgccCACAGCAGCGGCGAG |
|  | 236 | gtcgccCACAGCAGCGGCGAC |
|  | 237 | GGAGGCATGAACTCCTCGT |
| **4736631** | 271 | gttgccgCACGATCAGACCGGCAAC |
|  | 272 | attgccgCACGATCAGACCGGCAAT |
|  | 273 | CCTCGATGTTCTGCAGTCAG |
| **5611987** | 313 | cagatacaccTCTCGCAGCTGGTGTATCTG |
|  | 314 | tagatacaccGTCTCGCAGCTGGTGTATCTA |
|  | 315 | AGTCCGTCCACTTCCCATC |
| **479253** | 334 | ctcgcGTTGAGGCCCGCGAG |
|  | 335 | gtcgcgTTGAGGCCCGCGAC |
|  | 336 | ACGTTGCCGATGCTGTTAC |
| **2208216** | 379 | tcccCGCCGGTCTGGGGA |
|  | 380 | gcccctCGCCGGTCTGGGGC |
|  | 381 | ATGGGGGATGCACTACTGG |
| **2343185** | 385 | agcgCCCAGCGATTGCGCT |
|  | 386 | ggcgctTCCCAGCGATTGCGCC |
|  | 387 | CACAGGTGCGGTCAAGG |
| **3730244** | 430 | tacatgacacGCCTGCATGAGTGTCATGTA |
|  | 431 | aacatgacaGCCTGCATGAGTGTCATGTT |
|  | 432 | TCGAACACGTGAGCGACTAC |
| **3786552** | 433 | gcgccCCAGCACATTTTCTTGGCGC |
|  | 434 | acgccaaCAGCACATTTTCTTGGCGT |
|  | 435 | GCTTTGATGATGAACTCGGC |
| **753327** | 73 | cgggtgCGCCGATTACACCCG |
|  | 74 | tgggtgCCGCCGATTACACCCA |
|  | 75 | GTACCACCACAGCGATTACG |
| **1755920** | 118 | aggtccaaCGCACAACCTTTGGACCT |
|  | 119 | gggtcCGCACAACCTTTGGACCC |
|  | 120 | CACATCGCCGAGACCAG |
| **3714609** | 427 | ccgggATGCCGCCGAACCCGG |
|  | 428 | gcgggATGCCGCCGAACCCGC |
|  | 429 | CCAACCGTCAGCTTCTTTCA |
| **1718395** | 499 | CAGGAAGTGttgcgcagatacacttcctg |
|  | 500 | GAGGAAGTGttgcgcagatacacttcctc |
|  | 501 | gctcgaactcgatgaccag |
| **4342676** | 502 | GGTTGCTgttccatcgccagcaacc |
|  | 503 | TGTTGCTgttccatcgccagcaaca |
|  | 504 | atgccaagttcggcgagt |
| **5558163** | 505 | GCGTAAAGGGctgctcggcctttacgc |
|  | 506 | TCGTAAAGGGctgctcggcctttacga |
|  | 507 | cggcgtagtcggaatcac |
| **1425859** | 508 | GGTGTGCgccttcacggcacacc |
|  | 509 | AGTGTGCggccttcacggcacact |
|  | 510 | ggaatttgcgatcggttaga |
| **5382180** | 511 | ACCTATTTCGcgcgcccgaaataggt |
|  | 512 | GCCTATTTCGCgcgcccgaaataggc |
|  | 513 | Agcaccccgttgtacagg |
| **2056416** | 514 | TAATGCGGagccggccgcatta |
|  | 515 | CAATGCGCgccggccgcattg |
|  | 516 | gacgctcgcattgttcg |
| **749081** | 517 | GCATCTGGcgaagtggcccagatgc |
|  | 518 | ACATCTGGGgtcgaagtggcccagatgt |
|  | 519 | ccctgctggaagtcatcg |
| **1180129** | 520 | CGATGATGGGcaagtccgccatcatcg |
|  | 521 | TGATGATGGGccaagtccgccatcatca |
|  | 522 | cggcccgacggatt |
| **2041755** | 523 | TCTGACCCagagctgtatgtagagggtcaga |
|  | 524 | CCTGACCCgagctgtatgtagagggtcagg |
|  | 525 | accccagcgacaccact |
| **4368720** | 529 | GTTTTCCCTATCagcaccccgatagggaaaac |
|  | 530 | CTTTTCCCTATCagcaccccgatagggaaaag |
|  | 531 | acatatggaactcggcatcg |
| **4788987** | 532 | AAGGTATCCCgtcgccgtcggatacctt |
|  | 533 | GAGGTATCCCgtcgccgtcggatacctc |
|  | 534 | gactcgaagccccatcatc |
| **4921426** | 535 | ATAAATGCTTCaaggcggggaagcatttat |
|  | 536 | GTAAATGCTTCaggcggggaagcatttac |
|  | 537 | acgctcgcctccagctt |
| **2348891** | 541 | tccgcGCATATATCTTCGGCGGA |
|  | 542 | gccgcGGCATATATCTTCGGCGGC |
|  | 543 | GATCCGGGAAGCTTTTGTC |
| **5132618** | 556 | gtcgcgCAGGGCACCGCGAC |
|  | 557 | atcgcgCAGGGCACCGCGAT |
|  | 558 | TGCAATACACCGAGGAGATG |
| **3993860** | 565 | CCCGCgtccacgacgagcggg |
|  | 566 | TCCGCgtccacgacgagcgga |
|  | 567 | catccaggcagccaagac |
| **4260203** | 568 | ATCAAACCccacgacatcgggtttgat |
|  | 569 | GTCAAACCCcacgacatcgggtttgac |
|  | 570 | agctgctcgatctggtcc |
| **483854** | 577 | CGTGTCCctgccgtcggacacg |
|  | 578 | GGTGTCCtgccgtcggacacc |
|  | 579 | gcagatgcccgacctgt |
| **487781** | 580 | CGAAGTTTGgagacgacgaccaaacttcg |
|  | 581 | AGAAGTTTGGtgagacgacgaccaaacttct |
|  | 582 | actcgagaaagatcggcgta |
| **1483023** | 586 | AGTTAGCGAatgactcatcgtcgctaact |
|  | 587 | GGTTAGCGAatgactcatcgtcgctaacc |
|  | 588 | atgttgtagacccgcacctc |
| **4156440** | 589 | ACAGGCAggggaagcgtgcctgt |
|  | 590 | GCAGGCcggaagcgtgcctgc |
|  | 591 | gaccgattcacgtcggag |
| **4870071** | 595 | CGCCAAActagccttgtttggcg |
|  | 596 | TGCCAAActagccttgtttggca |
|  | 597 | aacgacgcgtattcgtttct |
| **348685** | 598 | CTGCCGacgttggccttcggcag |
|  | 599 | TTGCCGacgttggccttcggcaa |
|  | 600 | cgagccggttggacac |
| **3880170** | 601 | TGGTGCggtccagatagggcacca |
|  | 602 | CGGTGCggtccagatagggcaccg |
|  | 603 | cgcacgggccaaaa |
| **4028025** | 604 | GTCCGGcgctggcctaccggac |
|  | 605 | ATCCGGcgctggcctaccggat |
|  | 606 | ctgtggtggcgttgagc |
